# Adaptation to Resistance Training Is Associated with Higher Phagocytic (but Not Oxidative) Activity in Neutrophils of Older Women

**DOI:** 10.1155/2015/724982

**Published:** 2015-10-07

**Authors:** João Bartholomeu-Neto, Ciro José Brito, Otávio Toledo Nóbrega, Vinícius Carolino Sousa, Juliana Oliveira Toledo, Roberta Silva Paula, David Junger Fonseca Alves, Aparecido Pimentel Ferreira, Clayton Franco Moraes, Cláudio Córdova

**Affiliations:** ^1^Catholic University of Brasília, 71966-700 Brasília, DF, Brazil; ^2^Federal University of Juiz de Fora, 36036-330 Governador Valadares, MG, Brazil; ^3^University of Brasília, 70910-900 Brasília, DF, Brazil; ^4^Paulista University, 70390-130 Brasília, DF, Brazil

## Abstract

Failure in antimicrobial activity contributes to high morbidity and mortality in the geriatric population. Little is known about the potential effect of resistance training (RT) on the functional properties of the innate immunity. This study aimed to investigate the influence of long-term RT on the endocytic and oxidative activities of neutrophils and monocytes in healthy older women. Our results indicate that the phagocytosis index (PhI) of neutrophils (but not of monocytes) in the RT-adapted group was significantly higher (*P* < 0.001; effect size, (*d*) = 0.90, 95% CI: [0.75–1.04]) compared to that in sedentary subjects. In contrast, the oxidative activity of either neutrophils or monocytes was not significantly influenced by RT. Also, total energy and carbohydrate intake as well as serum IL6 levels had a significant influence on the phagocytic activity of neutrophils (*P* = 0.04), being considered in the model. Multivariate regression identified the physical condition of the subject (*β* = 0.425; *P* = 0.01) as a significant predictor of PhI. In conclusion, circulating neutrophils of older women adapted to a long-term RT program expressed higher phagocytic activity.

## 1. Introduction

The cascade of biological events that makes up the innate defense against infectious agents is a vital part of the immune system. Typically, this process is characterized by acute response triggered by the rapid increase in circulating inflammatory mediators [1]. However, during immunosenescence, there is complex remodeling of the immune system, characterized by exacerbation of the basal, possibly nonimmunologically derived profile of proinflammatory mediators and of reactive oxygen species, a phenomenon known as* inflammaging* [2]. This scenario is more evident after the fourth decade of life, when increased susceptibility to cancer, infections, and metabolic disorders is observed [3, 4].

Although the effects of aging on the innate immune system are not fully elucidated [5–7], it is assumed that this senescence-associated, subclinical inflammatory, and oxidative processes constitute a compensatory mechanism due to the decline of the endocytic capacity and reduced production of superoxides by phagocytic elements, among other age-triggered flaws of the immune function. Failure in neutrophil properties has been postulated as an important predictor of morbidity and mortality in the geriatric population [5].

Further studies aimed at exploring nonpharmacological interventions with potential to reverse the detrimental aspects of immunosenescence should be carried out [8, 9]. Although results already in the literature disclose benefits of resistance training (RT) on the systemic proinflammatory milieu of elderly subjects [10–12], little is known about the influence of RT on the functional response of cells that form the first line of defense of the immune system, particularly in this age stratum. This study aimed to investigate the influence of long-term RT on the phagocytic and oxidative activities of neutrophils and monocytes in apparently healthy older women. The intake of the main macronutrients and the circulating levels of important serum mediators of immunosenescence and endocrinosenescence were taken as potential confounding factors and considered herein. Our hypothesis is that chronic physiological adaptations induced by RT can modulate the endocytic and oxidative capacities of peripheral phagocytes in older women.

## 2. Materials and Methods

### 2.1. Participants

**Figure 1:**
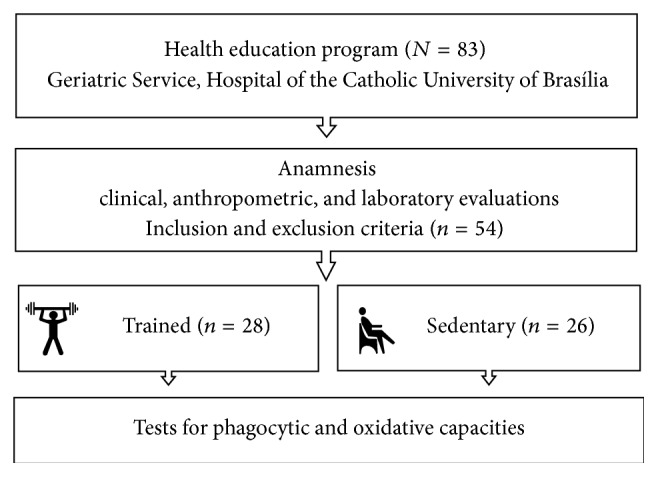
Procedures for sample selection and data collection.

The participants of this study were elderly women living in the community regularly followed by health education programs for prevention of chronic disorders developed by the Geriatric Service of the Catholic University of Brasilia Hospital, Brazil, known as the Prognosis and Therapeutics in Geriatrics (ProTeGer) study [13, 14]. After inspection of medical records, patients were excluded due to the following: uncontrolled type 2 diabetes, obesity, having smoked > 100 cigarettes over a lifetime, and/or having regularly consumed one dose (12 g) of alcohol per week or more over the last 12 months. Other exclusion criteria were use of immunomodulatory drugs or presence of neoplastic processes, acute infectious, and/or inflammatory signs at the time of clinical evaluations.  Figure 1 presents the chart of procedures adopted during this study.

The Lipschitz equation was used to determine the body mass index (BMI) [15]. The waist-to-hip ratio (WHR) was calculated based on waist circumference measurements at the midpoint between the last rib and the iliac crest and hip circumference in the greater trochanter. Body mass was measured with participants barefoot and wearing light clothing on a calibrated scale with 100 g accuracy (Filizola, São Paulo, SP, Brazil). Height was measured with stadiometer with accuracy of 1 cm. Body composition measurements were performed at the Laboratory of Image of the Institution using dual energy X-ray absorptiometry (DXA; Lunar DPX-IQ model, software version 4.7e, Lunar Radiation Corp., Madison, WI, USA), with individuals in the supine position on a horizontal platform, with slightly apart and relaxed legs, arms at body sides, and palms down. The software provided fat and fat-free masses. In addition to absolute masses, relative fat and fat-free measures were obtained with adjustment to height (kg/m^2^). The equipment was calibrated as recommended by the manufacturer. All examinations were performed by the same trained researcher.

### 2.2. Nutritional Assessment

Dietary data were determined based on food records of 3 days (2 working days and 1 day in the weekend), which is as accurate as records of 4 or 7 days [16, 17]. The food record was completed at home with patients being instructed to record food consumption in terms of number and size of portions. Values for each individual patient were expressed as the average intake of the 3 days reported. To ensure completion of food records, the staff of nutritionists provided personal or telephone assistance. The forms were returned during a clinical interview in which the amounts and types of foods were reviewed. Dietary contents were calculated using the Diet-Pro software, version 4.0 (A.S. Sistemas, Viçosa, MG, Brazil), configured for international food tables and complemented with a table for local food products [18]. Dietary intake of total protein, carbohydrates, and lipids was expressed as percentage of total energy and included in the analysis as continuous variables.

### 2.3. Resistance Training Prescription

For the RT prescription, the 1-maximum-repetition (MR) percentage was obtained according to methodology described by Kraemer et al. [19]. The group under RT was composed of volunteer women who have attended a moderate-intensity physical training (70% of 1 MR) consisting of exercise sessions three times a week on alternate days for 8.6 ± 0.3 months, with three sets of 12 repetitions per exercise at moderate speed and 1 minute of rest between sets. Each 50-minute session included nine exercises: horizontal leg press, knee extension, knee flexion, bench press, triceps extension in the pulley, biceps curling, seated rowing, plantar flexion, and abdominals. Whenever one participant was able to perform 13 repetitions or more in the third series of a given movement in the last weekday of training, loads were increased [20]. All equipment was manufactured by Righetto (Campinas, SP, Brazil), and warm-up exercises for 10 min preceded each training session.

All subjects were sedentary at baseline. The control group consisted of women who remained sedentary and participated during nine months in occupational activities not related to physical activity offered by the health education program. Attendance to either physical or occupational activities was entirely voluntary and freely chosen by each older-woman, as long as the three-session-per-week regimen was fulfilled when inscribed in RT-training. The study protocol was approved by the Research Ethics Committee of the institution and conducted in accordance with the Declaration of Helsinki.

### 2.4. Total and Differential Count of Leukocytes

Blood samples were diluted at 1 : 20 in Turk's solution. Leukocytes were counted in a Neubauer chamber (Labex, Aparecida de Goiânia, GO, Brazil) with concentration (cells/mm^3^) determined under optical microscopy by the equation [((*Q*1 + *Q*2 + *Q*3 + *Q*4)/4) × 200], with *Qn* as the number of cells in the quadrant *n* of the chamber and 200 as the product of 10 (conversion factor to 1 mm^3^, count area depth) and 20 (dilution factor). For differential leukocyte count, 100 cells in randomly distributed fields were considered.

### 2.5. Analysis of Serum Mediators

For measurement of immune mediators, blood samples were collected in the morning (8:00 to 9:30 a.m.) in tubes without endotoxin. For RT-adapted individuals, samples were collected at the end of the intervention period, along with samples from control subjects. Serum was separated within 1 hour after collection and stored at −80°C until processing day. Samples were analyzed using the enzyme-linked immunosorbent assay method (ELISA) with kits specific for interleukin 6 (IL6) and for tumor necrosis factor-alpha (TNF*α*) (eBioscience, San Diego, CA). Serum IGF1 levels were determined using automated Immulite 2000 Siemens system (Los Angeles, CA, USA). All samples were analyzed in duplicate.

### 2.6. Phagocytic Capacity Test

The endocytic capacity of circulating monocytes and neutrophils was tested* in vitro* by a* Saccharomyces cerevisiae* phagocytosis assay adapted from Muniz-Junqueira et al. [21]. Briefly, blood samples (40 *μ*L) were added in duplicate onto glass slides with 7 mm wide excavations and incubated in humid chamber for 45 min at 37°C. Then, slides were washed with PBS solution, pH 7.2 at 37°C. Adherent cells were incubated with 2.5 × 10^5^ yeasts in 20 *μ*L of Hanks-Tris solution (Sigma, St. Louis, MO, USA), pH 7.2, with 10% fetal bovine serum (FBS) (Cultilab, Campinas, Brazil). After 30 min of incubation in humid chamber at 37°C, slides were rinsed with PBS solution at 37°C, washed with Hanks-Tris solution with 30% FBS at 37°C, fixed with methanol, and stained with Giemsa solution. The number of* S. cerevisiae* phagocytosed by 200 monocytes or 200 neutrophils in individual preparations was evaluated by optical microscopy. The fields used for counting were randomly selected and all monocytes or neutrophils found were examined. The phagocytosis index (PhI) was calculated as the product of the following factors: (a) average number of* S. cerevisiae* phagocytosed by neutrophils or monocytes and (b) proportion of neutrophils or monocytes involved in the phagocytosis.

Yeast suspension was prepared following the method described by Lachmann and Hobart [22]. Briefly, 50 g tablet of fresh yeast (Fleischmann) was dissolved in 220 mL of a 0.05 M isotonic, phosphate buffered saline solution (PBS), pH 7.2, autoclaved at 120°C for 30 minutes, and then washed for 3 times in PBS by centrifugation at 4,000 rpm for 5 min. This procedure was repeated to obtain a clear supernatant. The sediment was resuspended in 28 mL of PBS containing 2-mercaptoethanol at 0.1 M. After two hours of stirring at 37°C, the suspension was washed again and the sediment was resuspended in 55 mL in PBS solution containing 0.02 M iodoacetamide. After further stirring at 37°C for two hours, the suspension was washed three times, resuspended in 220 mL of PBS, autoclaved once more, and washed with PBS by centrifugation to obtain a clear supernatant, being finally resuspended in 110 mL of Veronal buffer of pH 7.2 containing sodium azide (200 mg/L). The yeast suspension was stored at 4°C for use for a period not exceeding one week after its production. Prior to each assay, the yeast suspension was washed in PBS, suspended in equal volume of Hanks-Tris solution, quantified, and used immediately.

### 2.7. Oxidative Capacity Test

The oxidative capacity test was adapted from technique described by Park et al. [23] to evaluate the production of reactive oxygen species based on the principle of reduction of nitroblue tetrazolium (NBT) up to its insoluble form (formazan). Briefly, phagocytes adhered to excavation, as previously described, were incubated with Hanks-Tris solution containing 0.05% of NBT for 20 min at 37°C in humid chamber. The slides were rinsed, fixed with methanol, and stained with aqueous solution containing 1.4% safranin and 28.6% glycerol. The proportion of phagocytes that reduced NBT in their cytoplasm was determined by counting 200 neutrophils and 200 monocytes found. Positive, NBT-reducing phagocytes were those that presented the typical blackish-blue cytoplasmic precipitate that is compatible with formazan formation.

The phagocytic and oxidative capacities were intended to be assessed simultaneously and for 2 or 3 subjects at once. After assaying all subjects, our first-round success rate scored at 90%, with minimum slide quality achieved whenever distinction of leucocytes and identification of yeasts were possible by visual inspection. Blanks were filled by new invitations for blood draw until data was completed for the whole sample. All tests were performed by the same trained biologist.

### 2.8. Statistical Analysis

Data analysis was performed using the Statistical Package for Social Sciences (SPSS) for Windows (version 8.0). The Kolmogorov-Smirnov test was used to test the data normality. To obtain a close-to-normal distribution, data were logarithmically transformed (log_10_), whenever necessary. Student's *t*-test for independent samples was used to compare groups in terms of body composition, calorie intake, age, white blood cell count, and levels of proinflammatory mediators. Analysis of covariance (ANCOVA) was performed using both groups (RT versus sedentary) as independent variables to investigate possible differences induced by exercise on the phagocytic activity, with IL6, BMI, and caloric intake measures as covariant. Associations of body composition, total protein, carbohydrates, lipids, IL6, TNF*α*, and IGF1 measurements with phagocytosis of monocytes and neutrophils were analyzed using Pearson's product-moment correlation coefficient. Where appropriate, the effect size (*d*) and confidence interval (95% CI = *d* ± *Z*_0.025_ *S*_*d*_) were estimated. Multivariate linear regression with a stepwise variable selection was used in order to test the relationship between phagocytic activity and potential predictors of the regression model. Assumptions of normality, homogeneity, and independence of wastes were investigated, the former two by visual inspection and the latter by the Durbin-Watson statistics (*d* = 1.7). Variance inflation factor was used for the diagnosis of possible collinearity among variables (VIF's < 1.3). *P* < 0.05 was considered statistically significant in bilateral tests.

## 3. Results

After applying the exclusion criteria, 54 older women (71.3 ± 6.3 years) composed two groups, 28 in the RT-adapted subset and 26 as a sedentary counterpart. Participants' characteristics are described in Table 1. When compared to the sedentary group, women adapted to RT exhibited reduced average values for total caloric intake (~23%) and systolic blood pressure (~6%). Similarly, reduced circulating levels of proinflammatory biomarkers log_10_ IL6 (~30%) and log_10_ TNF*α* (~22%) were observed in the RT-adapted group.

Concerning phagocytosis by monocytes and neutrophils, comparisons between groups are shown in Table 2. Increased phagocytic capacity of neutrophils was observed among RT-adapted participants compared to the sedentary group, represented by both greater proportion of* S. cerevisiae* captured (~31%; *P* < 0.001) and a higher frequency of phagocyting cells (~18%, *P* = 0.033). Thus, the PhI of neutrophils was significantly higher in the RT-adapted group (~57%; *P* < 0.001, *d* = 0.90, 95% CI: [0.75–1.04]) compared to the group without exercise. Among covariates, total energy intake and the intake of carbohydrates had a significant influence on the phagocytic activity of neutrophils (*F*(1.53) = 3.67; *P* = 0.04), being necessarily considered to be covariables in our models. In contrast, no statistically significant differences in the phagocytic ability of monocytes between groups were observed. The production of reactive oxygen species in neutrophils and monocytes, as assessed by the proportion of cells that promoted NBT reduction, was not significantly influenced by RT.

**Figure 2:**
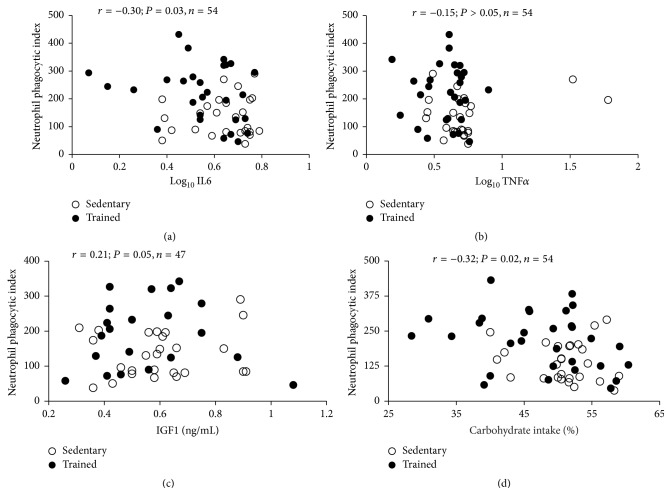
Correlations of log_10_ IL6 (a), log_10_ TNF*α* (b), IGF1 (c), and total carbohydrate intake (d) with neutrophil phagocytic index. *r* = Pearson's correlation coefficient.

Results of correlation tests with inclusion of all participants showed a significant negative association of the neutrophils' PhI with circulating levels of IL6 (*r* = −0.29; *P* = 0.03) and the intake of total carbohydrates (*r* = −0.32; *P* = 0.01) (Figure 2). There were no significant associations between PhI and circulating levels of IGF1 and TNF*α*. Multivariate regression analysis identified the physical condition of subjects (RT-adapted or sedentary) as the most significant variable to predict PhI (*β* = 0.425; *P* = 0.01). Log_10_ IL6 and caloric intake were excluded from the model.

## 4. Discussion

In the field of immunology, it is generally recognized that rapid activation and recruitment of neutrophils reflect the integrity of important signaling pathways involved with the initiation of immune responses, to result in protective inflammation [24, 25]. The main findings of this study are consistent with a higher phagocytic activity of circulating neutrophils in older women adapted to a long-term RT program in relation to the sedentary group. Since the disparity in PhI between groups was not mitigated with statistical control for potentially confounding factors (as nutritional profile, anthropometry, and cytokine levels), it is suggested that the benefits of RT may be the result of independent physiological mechanisms (e.g., hormonal), as advocated by evidence presented elsewhere [26].

Although little is known about the influence of exercise on the function of neutrophilic polymorphonuclear leukocytes in the geriatric populations [27], our results corroborate study that found lower phagocytic activity of neutrophils in sedentary elderly subjects compared to those physically active [28]. Study including middle-aged recreational soccer players showed higher phagocytic activity compared to sedentary subjects [29]. Although the cross-sectional nature of our study does not allow the establishment of causal relationship, these results suggest that the expected decline of the phagocytic response with aging [3, 5] may be at least partially reversed by adaptations induced by RT in populations under high risk of functional capacity decay.

On what concerns production of reactive oxygen species, our results did not show significant differences between groups, corroborating findings in different population strata and sportive modalities [29–31]. It has been well established that neutrophils may exhibit impaired capacity to produce superoxides in geriatric subjects [32, 33]. We do not rule out that this result can, at least in part, be influenced by the expected age-related reduction of dehydroepiandrosterone (DHEA) to levels that may restrict potential benefits from physical exercise, since this circulating steroid has proven to be important to the synthesis of superoxides in neutrophils [34].

Regarding the fact that the monocyte functions have not been influenced by RT, it is likely that the monocytes/macrophages system may depend on its developmental state to benefit from endocrine mediators promoted by physical exercise [35]. Monocytes are a class of relatively immature circulating phagocytes that demand migration to tissues as a requirement for full cell specialization. It is plausible that different phagocytic indexes between monocytes and neutrophils arise from this relative immaturity of monocytic types or that the adaptations induced by our intervention have been insufficient to promote important endocrine changes to the point of favoring direct activation of monocytes. In line with this, we do not rule out the possibility that the sample size was insufficient to obtain a significant effect on monocytes' indexes.

The analysis of the inflammatory profile of the subjects in this study corroborates evidence from literature that associates increased IL6 levels with an age-dependent dysregulation of the innate immune system [34]. Regarding the circulating TNF*α* level, the results showed no significant associations with the phagocytic activity. This finding is in line with studies in human models that support the concept that TNF*α* is not a robust biomarker that reflects systemic inflammation in sedentary populations when compared to abnormal endocrine [36] and tissue [37] levels of IL6. Therefore, it is likely that the serum IL6 is a more robust predictor of physical condition in apparently healthy yet sarcopenia-prone geriatric populations, due to evidence that IL6 (but not TNF*α*) tends to be actively produced by the exercising muscle [38, 39].

Factors extrinsic to the immune system are implicated in the imbalance of the innate immune system with aging. Phagocytes are exposed to a variety of extraimmune agents (hormonal, mainly), which may affect their functional phenotype [7] by changes in intracellular signaling cascades [33], for instance. In our conditions, the phagocytic activity of neutrophils was not influenced by the circulating levels of insulin-like growth factor-1 (IGF1). Despite evidence that RT produces acute increases in serum IGF1 and in IGF1R on circulating monocytes [40], few studies have investigated the influence of RT on levels of this potent anabolic factor [41–43], with inconsistent results [44]. It is likely that the different protocols analyzed and the small frequency of postexercise assessments could explain these results or that neuroendocrine (e.g., catecholamines) signals are more effective modulators of phagocytic functions than the GH axis [26].

Despite the fact that total energy and carbohydrate intakes produced significant impact on the phagocytic response of neutrophils, it is important to observe that statistical control for such covariable did not suppress the association found between PhI and the physical status (RT-adapted or sedentary) of the older women investigated. The women in the RT group exhibited lower total caloric intake compared to sedentary subjects probably due to the reduced carbohydrate intake. Even though the authors consider this result to be important since the amount of nutrients ingested has been associated with changes in both cellular and humoral responses [10, 45], no explanation can be provided at this point for such disparity in the pattern of energy consumption displayed by the subjects. But we suggest that calorie intake should be controlled in studies investigating the relationship between exercise/physical activity and phagocytic response.

The cross-sectional nature of this study is a limitation, so the results should be interpreted with care. Prevalence bias cannot be ruled out, since an uneven distribution of preexisting health conditions not considered in the setting up of the groups may have resulted in more elderly women with an exceptional immune status to be included in the RT-trained group, for instance. On their behalf, the authors sustain that their analyses were designed with the purpose of controlling potential confounding variables associated with the effects of acute infectious processes, use of immunomodulatory drugs, systemic levels of relevant immune mediators, and nutritional factors prior to the phagocytic and oxidative assays.

## 5. Conclusion

The main findings of this study suggest that circulating neutrophils (but not monocytes) in older women adapted to long-term RT program bear higher phagocytic capability in relation to those of sedentary counterparts. There was no influence of RT on the oxidative activity of either neutrophils or monocytes. We suggest that the nutritional parameters and circulating immunomodulators also correlate with the phagocytic index of neutrophils and therefore must be controlled in studies addressing the relationship between exercise/physical activity and the phagocytic response in populations susceptible to significant weaknesses.

## Figures and Tables

**Table 1:** Characteristics of the sample.

	Sedentary (*n* = 26)	Trained (*n* = 28)	*P* ^*∗*^
Age, years	72.0 ± 6.9	70.6 ± 5.7	0.406
BMI, kg/m^2^	27.9 ± 4.4	29.5 ± 5.5	0.223
Total fat-free mass, kg	36.5 ± 2.4	39.1 ± 5.9	0.040
Relative fat-free mass, kg/height^2^	15.8 ± 0.9	16.4 ± 1.8	0.090
Total fat mass, kg	24.9 ± 7.2	25.8 ± 8.1	0.121
Relative fat mass, kg/height^2^	11.8 ± 3.1	12.9 ± 3.8	0.208
Waist circumference, cm	97.4 ± 9.2	99.8 ± 12.2	0.406
Calorie intake, 10^3^ kcal^*∗*^	2.2 ± 0.6	1.7 ± 0.5	0.001
Total carbohydrate intake, %	50.1 ± 4.9	47.4 ± 8.3	0.050
Total lipid intake, %	34.1 ± 4.9	36.0 ± 7.6	0.129
Total protein intake, %	14.7 ± 3.8	15.5 ± 3.5	0.424
Systolic blood pressure, mmHg	134.3 ± 13.0	125.8 ± 14.1	0.022
Diastolic blood pressure, mm Hg	81.9 ± 9.9	80.8 ± 9.5	0.670
IGF1, ng/mL	0.60 ± 0.17	0.56 ± 0.19	0.410
Leukocyte numbers, mm^3^	5.2 ± 2.1	5.7 ± 2.2	0.401
Neutrophils, %	55.4 ± 11.7	53.2 ± 9.7	0.449
Segmented	53.4 ± 11.3	52.1 ± 9.5	—
Banded	2.0 ± 1.5	1.1 ± 1.4	—
Monocytes, %	3.9 ± 2.2	3.8 ± 2.8	0.910
Lymphocytes, %	37.9 ± 10.4	39.8 ± 10.1	0.494
Eosinophils, %	2.7 ± 2.6	3.1 ± 2.7	0.608
TNF*α* pg/mL	0.73 ± 0.29	0.57 ± 0.20	0.036
IL6 pg/mL^*∗*^	0.63 ± 0.12	0.44 ± 0.36	0.002

Values are expressed as mean ± standard deviation. ^*∗*^Statistical difference *P* < 0.050.

**Table 2:** Phagocytic and oxidative activities in the samples.

	Sedentary (*n* = 26)	Trained (*n* = 28)	*P* ^*∗*^
*S. cerevisiae* phagocyte/neutrophils, *n*	2.2 ± 0.5	2.9 ± 0.9	<0.001
*S. cerevisiae* phagocyte/monocytes, *n*	1.4 ± 0.2	1.5 ± 0.3	0.416
Neutrophils in phagocytosis, %	60.5 ± 19.6	71.2 ± 19.1	0.033
Monocytes in phagocytosis, %	40.4 ± 14.3	40.5 ± 10.6	0.868
Index of neutrophils phagocytosis	140.4 ± 69.5	221.2 ± 105.8	<0.001
Index of monocytes phagocytosis	53.8 ± 22.4	60.5 ± 18.2	0.160
Neutrophils reduced NBT, %	51.3 ± 23.0	45.3 ± 26.4	0.328
Monocytes reduced NBT, %	49.0 ± 19.6	46.8 ± 21.1	0.294

Values are expressed as mean ± standard deviation. ANCOVA adjusted for IL6, BMI, and calorie intake. ^*∗*^Statistical difference set at *P* < 0.05.
